# Usefulness of third‐generation narrow band imaging and texture and color enhancement imaging in improving visibility of superficial early gastric cancer: A study using color difference

**DOI:** 10.1002/deo2.186

**Published:** 2022-11-24

**Authors:** Azusa Kawasaki, Naohiro Yoshida, Hiroyoshi Nakanishi, Shigetsugu Tsuji, Kenichi Takemura, Hisashi Doyama

**Affiliations:** ^1^ Department of Gastroenterology Ishikawa Prefectural Central Hospital Ishikawa Japan

**Keywords:** color difference, early gastric cancer, image‐enhanced endoscopy, narrow band imaging, texture and color enhancement imaging

## Abstract

**Objectives:**

Overlooking early gastric cancer (EGC) during endoscopy is an issue to be resolved. Image‐enhanced endoscopy is expected to improve EGC detection. This study investigated the usefulness of third‐generation narrow band imaging (3G‐NBI) and texture and color enhancement imaging (TXI) in improving the visibility of EGC using the color difference between EGC and its surrounding gastric mucosa.

**Methods:**

In this retrospective observational study, we examined 51 superficial EGCs that underwent endoscopic submucosal dissection and were observed by all three methods: 3G‐NBI, TXI, and white light imaging (WLI). The primary endpoint was to compare the color difference of each method. For each EGC, we prepared one non‐magnifying image for each method so that the location and size of the lesion in each image were the same. The L*a*b* color space was used to evaluate the color values. When the color values of the cancerous lesion and its surrounding mucosa were (L*_c_, a*_c_, b*_c_) and (L*_s_, a*_s_, b*_s_), respectively, the color difference was defined to be [(L*_c_−L*_s_)^2^+(a*_c_−a*_s_)^2^+(b*_c_−b*_s_)^2^]^1/2^.

**Results:**

The median color difference was 9.2 (interquartile range, 5.3–15.7) in WLI, 13.5 (interquartile range, 9.4–19.5) in 3G‐NBI, and 15.3 (interquartile range, 9.1–22.1) in TXI. Statistically, the color difference was significantly larger in 3G‐NBI than in WLI (*p* < 0.001) and TXI compared with WLI (*p* < 0.001). However, there was no significant difference between 3G‐NBI and TXI (*p* = 0.330).

**Conclusions:**

Regarding color difference, both 3G‐NBI and TXI were estimated to be more useful than WLI in improving the visibility of superficial EGC.

## INTRODUCTION

Worldwide, gastric cancer is one of the most common cancers, with more than 1 million cases per year and 5.7% of all cancer diagnoses.^1^, ^2^ Its prognosis is poor with a 5‐year survival rate of 31% because most cases are already metastatic when diagnosed.^2^ Ratios of early gastric cancer (EGC) are high in gastric cancer detected by screening esophagogastroduodenoscopy.^3^ Moreover, the group of patients treated for endoscopically detected EGC had fewer deaths than the group that did not receive treatment.^4^ These findings suggest that endoscopic detection and treatment of EGC may reduce mortality from gastric cancer.^5^ White light imaging (WLI) is the most common method of endoscopic observation of the stomach^6^; however, even expert endoscopists can overlook EGC under standard WLI. The endoscopic abnormalities of EGC observed by WLI are often subtle and indistinguishable from benign inflammatory changes in the surrounding mucosa. Therefore, missing EGC by WLI is not uncommon, and a meta‐analysis article reports a rate of 9.4% missed ECGs (95% confidence interval 5.7%–13.1%).^7^

The usefulness of image‐enhanced endoscopy (IEE) for the detection of EGC has been studied.^8^ Narrow band imaging (NBI) is representative of IEE, and second‐generation NBI (2G‐NBI) produces higher‐quality images than first‐generation NBI owing to the improved NBI filter and a xenon lamp. However, 2G‐NBI did not increase the EGC detection rate over conventional WLI in a randomized controlled trial.^9^ In 2020, a new endoscopic system using five‐color light‐emitting diodes (LEDs) as a light source became available. This system allows the use of third‐generation NBI (3G‐NBI) and texture and color enhancement imaging (TXI) for detecting EGC. Regarding 3G‐NBI, no studies have examined its usefulness for detecting EGC. TXI has been reported to improve the visibility of EGC compared with WLI,^10^, ^11^ but there are no studies comparing TXI with other IEE.

This study aimed to simultaneously investigate the usefulness of 3G‐NBI and TXI in improving the visibility of superficial EGC by evaluating the color difference between the cancerous lesion and the surrounding gastric mucosa.

## METHODS

### Study design

This single‐center, retrospective observational study was conducted at Ishikawa Prefectural Central Hospital, a tertiary referral center in Japan. In accordance with the Declaration of Helsinki, the institutional review board of Ishikawa Prefectural Central Hospital approved this study (approval No.1966; June 7, 2022), and all subjects were provided the opportunity to opt out. Because there were no appropriate previous studies, we conducted this exploratory study with a limited target period instead of calculating the sample size.

### ECGs studied

**FIGURE 1:**
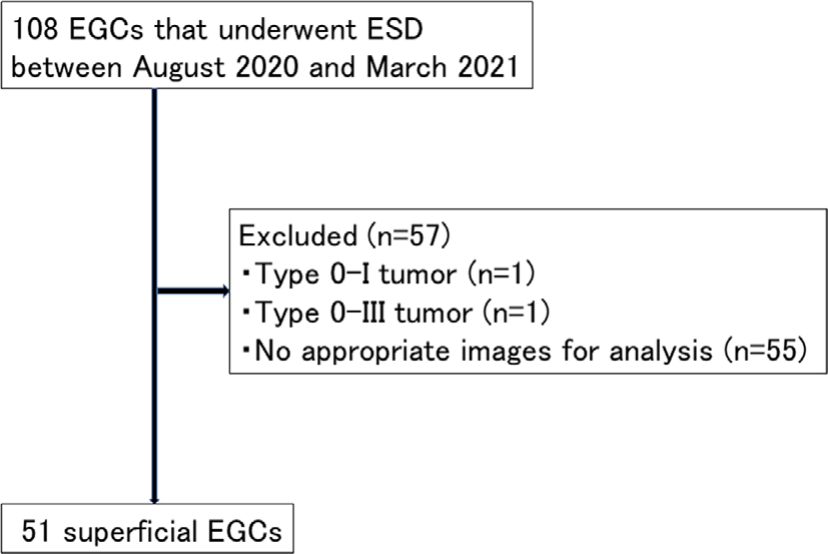
Flowchart of the study subjects. Note, 108 early gastric cancers (EGCs) underwent endoscopic submucosal dissection (ESD) between August 2020 and March 2021

Among 108 EGCs that underwent endoscopic submucosal dissection (ESD) between August 2020 and March 2021, we examined 51 superficial EGCs that were scrutinized by all three observation methods (3G‐NBI, TXI, and WLI) before ESD. Because the protruding type (0–I) and excavated type (0–III) are easy to recognize, the eligible macroscopic type was limited to the superficial type (0–IIa, 0–IIb, and 0–IIc) for this study. We excluded 1 type 0–I, 1 type 0–III, and 55 EGCs for which appropriate images could not be prepared for analysis (Figure 1).

### Instruments

An endoscopic system (EVIS X1) with a video processor (CV‐1500) was used in the study. This is a novel endoscopic system that uses LEDs in five colors as a light source and has an IEE called TXI in addition to 3G‐NBI. The video processor settings for structure enhancement were type B, level 6 for WLI, and type B, level 8 for NBI. TXI was used in mode 1. We used the GIF‐EZ1500, GIF‐XZ1200, or GIF‐H290Z gastrointestinal videoscope. These gastroscopes allow endoscopists to switch to 3G‐NBI or TXI by pressing a button on the gastroscope. All the above‐mentioned endoscopic instruments are manufactured by Olympus Corporation (Tokyo, Japan).

### 3G‐NBI

The NBI system has a dedicated, built‐in, narrow‐bandwidth filter in its light source, with central wavelengths of 415 nm and 540 nm and a bandwidth of 30 nm.^12^ Because hemoglobin absorbs this narrow‐band light, the microvascular architecture of the mucosal surface can be easily visualized. In contrast to 2G‐NBI, which uses a xenon lamp as the light source, we have defined 3G‐NBI as the NBI that uses a five‐color LED as the light source.

### Texture and color enhancement imaging

TXI is a new IEE modality.^13^ This is a white light‐based IEE that is designed to improve the dimensional characterization of subtle surface irregularities, enhance brightness in dark areas, and emphasize color changes.

TXI consists of six consecutive processes. A white light endoscopic image is separated into a detail layer and a base layer. The texture is highlighted by enhancing the detail layer. A dark area can be selectively brightened to adjust the brightness of the base layer. The two enhanced layers are merged to create a TXI as output. Additionally, the color tone of the output is augmented, amplifying the differences between color images, particularly between the bandwidths for white and red, by directly enhancing the color tone in the International Commission on Illumination (CIE) 1976 L∗a∗b∗space.^14^ TXI has two settings: TXI mode 1 (texture and brightness, and color enhancement) and TXI mode 2 (texture and brightness enhancement). We used TXI mode 1 in this study because previous studies suggested that TXI mode 1 is more useful than TXI mode 2 in the recognition of EGC.^10^, ^11^

### Evaluation of color difference

**FIGURE 2:**
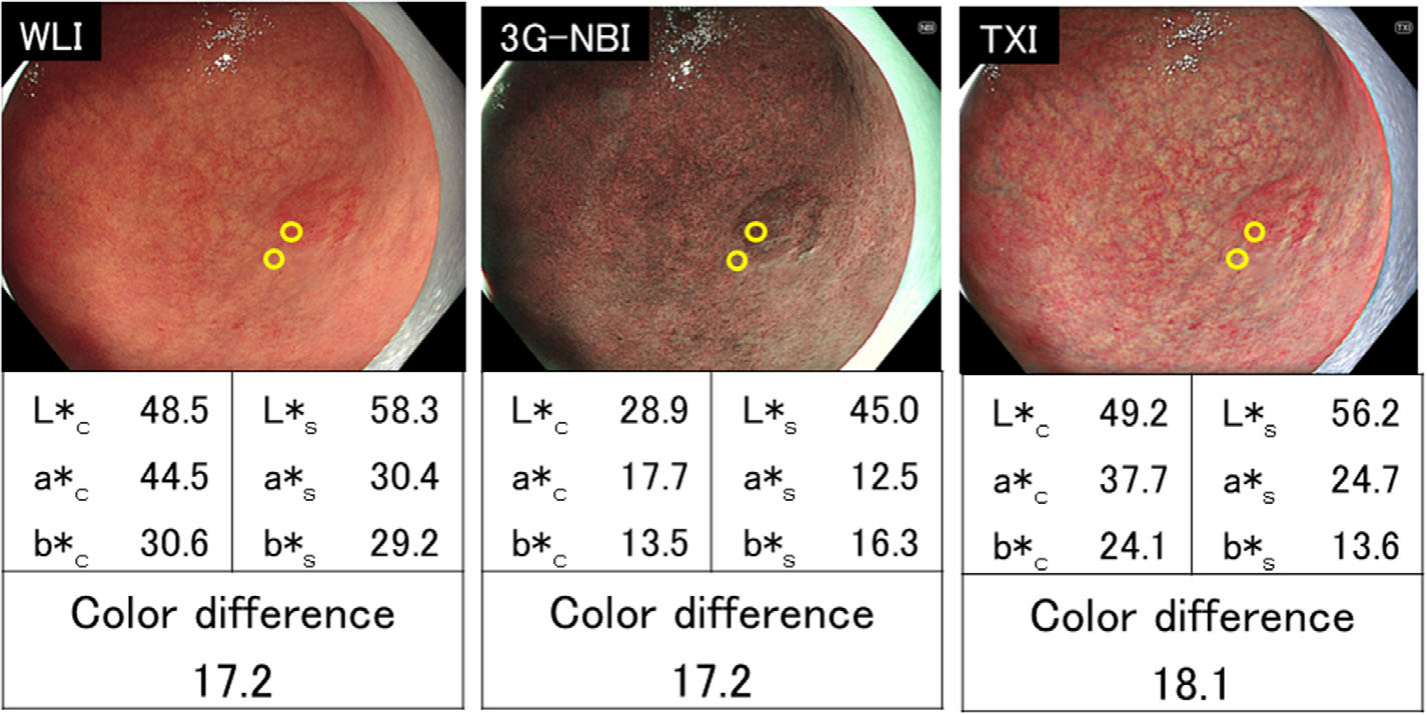
Representative color difference measurement. A depressed lesion (0‐IIc) in the middle third of the stomach was observed. The final histopathological diagnosis was well‐differentiated adenocarcinoma, confined to the mucosa. The color difference was 17.2 in white light imaging (WLI), 17.2 in third‐generation narrow band imaging (3G‐NBI), and 18.1 in texture and color enhancement imaging (TXI). When the color values (L*a*b*) of the region of interest at the edge of the cancerous lesion and its surrounding mucosa were represented as (L*_c_, a*_c_, b*_c_) and (L*_s_, a*_s_, b*_s_), respectively, the color difference was defined to be [(L*_c_−L*_s_)^2^+(a*_c_−a*_s_)^2^+(b*_c_−b*_s_)^2^]^1/2^

A representative case illustrating the evaluation of color difference is shown in Figure 2. For each lesion, we prepared one non‐magnifying image (1920 × 1080 pixels) for each method so that the location and size of the lesion in the three images (3G‐NBI, TXI, and WLI) were the same. Regarding image selection, firstly we extracted all non‐magnifying images in which the entire lesion was captured and the lesion was moderately extended. Then, we selected one set of three images from these extracted images, in which the lesion was most similarly captured in all three methods. A region of interest (ROI) was defined as a circle with a diameter of 24 pixels with reference to a previous study.^15^ One ROI was set for each cancerous lesion and the surrounding mucosa at the lesion boundary near the lens of the endoscope. The lesion boundaries in the images were determined based on the results of magnifying NBI and pathology obtained with ESD specimens. The CIE L*a*b* color space developed in 1976 was used to evaluate the hue. When the color values (L*a*b*) of the ROI at the edge of the cancerous lesion and its surrounding mucosa were represented as (L*_c_, a*_c_, b*_c_) and (L*_s_, a*_s_, b*_s_), respectively, their color difference was defined to be [(L*_c_−L*_s_)^2^+(a*_c_−a*_s_)^2^+(b*_c_−b*_s_)^2^]^1/2^. We compared the color differences obtained by the three observation methods. All calculations of color difference were performed using Image Processing and Analysis in Java software (ImageJ^16^). The color values were measured by a different person than the one who performed the statistical analysis for this study.

### Definition of EGCs

An EGC is defined as a tumor confined to the mucosa or submucosa (without regard to lymph node metastasis), and it was classified according to the Japanese Classification of Gastric Carcinoma.^17^ The depth of cancer was divided into the following two groups: T1a was considered to be a tumor confined to the mucosa, and T1b was considered to be a tumor confined to the submucosa. Malignant epithelial tumors, including papillary, tubular (well and moderately differentiated), poorly differentiated (solid and non‐solid type), signet‐ring cell, and mucinous, were diagnosed as gastric cancer. Histological types were divided into two categories: differentiated (papillary or tubular) and undifferentiated (poorly differentiated or signet‐ring cell or mucinous). Gross types were classified as 0‐IIa (superficial elevated), 0‐IIb (superficial flat), or 0‐IIc (superficial depressed). Tumor locations were anatomically classified into three portions, the upper, middle, and lower parts, by the lines connecting the trisected points on the lesser and greater curvatures of the stomach.

### Evaluation of background factors

The Kimura–Takemoto classification was used for the endoscopic diagnosis of gastric mucosal atrophy.^18^ In the Kyoto Classification of Gastritis developed in 2015,^19^ gastric mucosal atrophy was divided into three groups: none (C‐0 to C‐1), mild (C‐2 to C‐3), and severe (O‐1 to O‐3). In accordance with this classification, patients were divided into a non‐atrophic group (C‐0 to C‐1), and a mildly and severely atrophic group (C‐2 to O‐3) in this study. We also examined the gastric mucosal atrophy around the lesion, as it may affect the color difference.

*Helicobacter pylori* (*H. pylori*) infection status was divided into three groups: current infection, non‐infection, and past infection. Current infection was defined as positive by any of the following approaches: rapid urease test, microscopic examination, culture method, serum immunoglobulin G antibody testing (≥10 U/ml; *H. pylori* antibody kit; Special Reference Laboratories, Tokyo, Japan), urea breath test, or fecal *H. pylori* antigen measurement. Non‐infection was defined as a negative result for all tests performed in serum immunoglobulin G antibody testing (<3 U/ml), urea breath test, and fecal *H. pylori* antigen measurement, and a case that was endoscopically considered as uninfected using the Kyoto Classification of Gastritis.^19^ Finally, past infection was defined as that which did not fit either the current infection or non‐infection definitions.

### Outcomes

The primary endpoint was to compare the color difference in the three groups (WLI, 3G‐NBI, and TXI). The secondary endpoint was to compare color differences in the three groups for each location, macroscopic type, histological type, median tumor size, tumor depth, endoscopic gastric atrophy, *H. pylori* infection status, and gastrointestinal videoscope used.

### Statistical analysis

All statistical analyses were performed using EZR (Easy R; Saitama Medical Center, Jichi Medical University, Saitama, Japan), which is a graphical user interface for R (www.r‐project.org). More precisely, it is a modified version of R Commander, designed to add statistical functions frequently used in biostatistics.^20^ Continuous variables are presented as the median. A comparison of the color difference between the three modes was examined using the Wilcoxon signed‐rank test. The Bonferroni correction was performed on the primary endpoint, and a *p*‐value of less than 0.016 (=0.05/3) was considered statistically significant.

## RESULTS

The clinicopathological characteristics are shown in Table 1. There were 46 males (90.0%), with a median age of 75 (range 55–90). Most patients (96.0%) showed mild and severe endoscopic gastric atrophy and gastric mucosal atrophy around the lesion. Thirty‐nine patients (76.5%) had a current or past *H. pylori* infection, and two patients (3.9%) had non‐infection status. The predominant gastrointestinal videoscope used was GIF‐EZ1500 (54.9%) followed by GIF‐H290Z (25.5%). Eighty percent of lesions were located in the upper two‐thirds of the stomach. The predominant macroscopic type was type 0‐IIc (64.7%) followed by type 0–IIa (33.3%), and the median tumor size was 13 mm (range 3–41 mm). Most lesions (92.2%) were differentiated in the histological types and 88.2% were pT1a in the invasion depth. Biopsies were performed prior to observation in all but one lesion.

**TABLE 1:** Clinicopathological characteristics

Patient characteristics and instrument used	*N* = 51
Sex	
Male	46
Female	5
Median age, years (range)	75 (55–90)
Endoscopic gastric atrophy	
Mild and severe	49
None	2
*H. pylori* infection status	
Current	25
Non‐infection	2
Past	14
Unknown	10
Gastrointestinal videoscope	
GIF‐EZ1500	28
GIF‐XZ1200	10
GIF‐H290Z	13

Lesion characteristics	*N* = 51
Location	
Upper	20
Middle	20
Lower	10
Anastomosis	1
Macroscopic type	
0–IIa	17
0–IIb	1
0–IIc	33
Median tumor size, mm (range)	13 (3–41)
Histological type	
Differentiated	47
Undifferentiated	4
Invasion depth	
pT1a	45
pT1b	6
Atrophy around the lesion	
Presence	49
Absence	2

Abbreviation: *H. pylori, Helicobacter pylori*.

**FIGURE 3:**
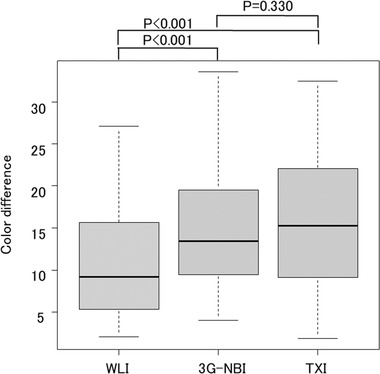
Comparison of color differences in the three groups: white light imaging (WLI), third‐generation narrow band imaging (3G‐NBI), and texture and color enhancement imaging (TXI). The color difference was significantly larger in 3G‐NBI than in WLI (13.5 vs. 9.2, respectively; *p* < 0.001) and significantly larger in TXI than in WLI (1.3 vs 9.2, *p* < 0.001). However, there was no significant difference between 3G‐NBI and TXI (*p* = 0.330)

The median color difference was 9.2 (interquartile range 5.3–15.7) in WLI, 13.5 (interquartile range 9.4–19.5) in 3G‐NBI, and 15.3 (interquartile range 9.1–22.1) in TXI. Statistically, the color difference was significantly larger in 3G‐NBI than in WLI (*p* < 0.001) and significantly larger in TXI than in WLI (*p* < 0.001). However, there was no significant difference between 3G‐NBI and TXI (*p* = 0.330; Figure 3).

**FIGURE 4:**
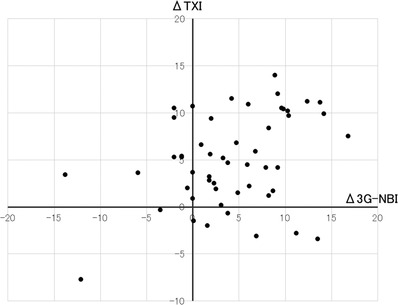
Color difference with respect to white light imaging (WLI) in each lesion. A third‐generation narrow band imaging (3G‐NBI) is defined as the difference in color between 3G‐NBI and WLI. A Δtexture and color enhancement imaging (TXI) is defined as the difference in the color difference between TXI and WLI

**FIGURE 5:**
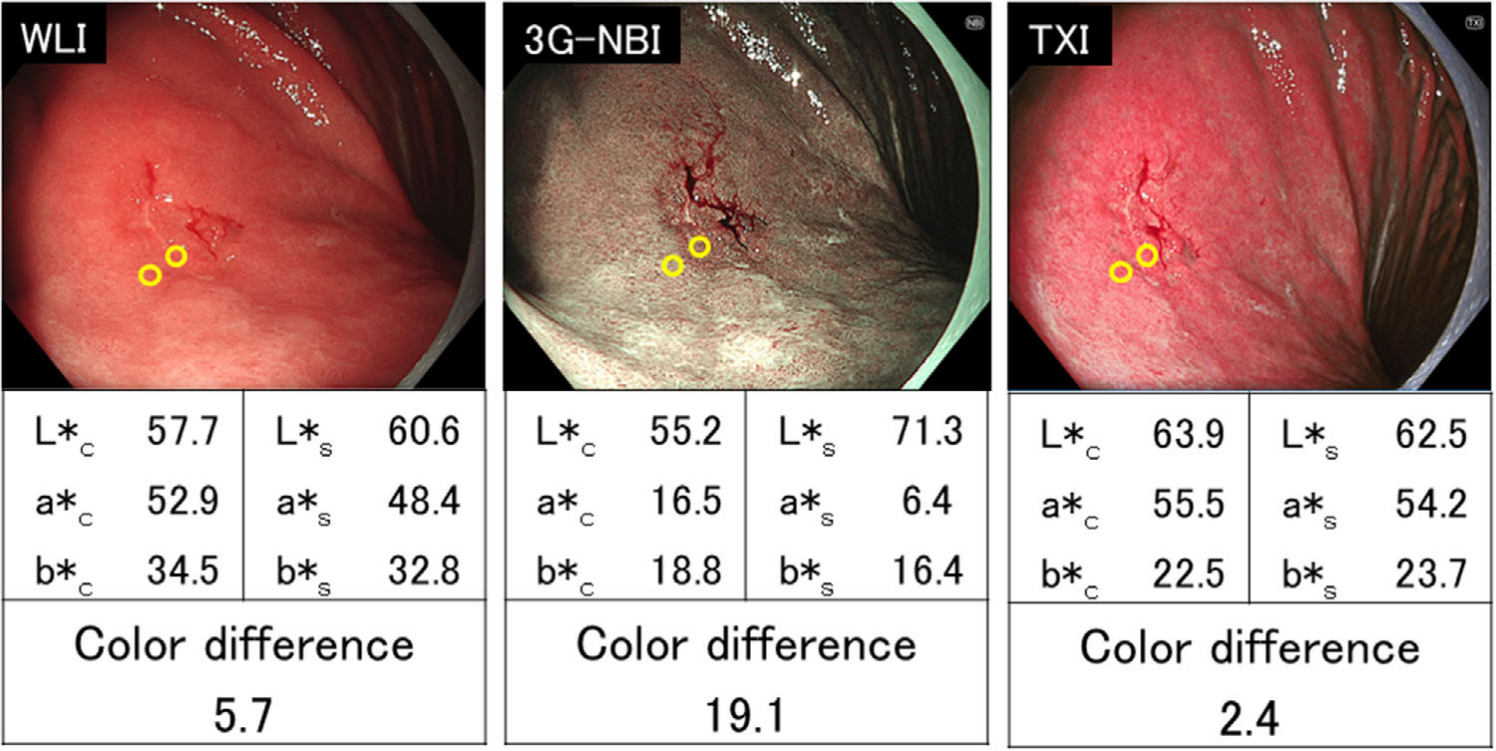
A representative case in which the color difference was larger in third‐generation narrow band imaging (3G‐NBI) and smaller in texture and color enhancement imaging (TXI) than in white light imaging (WLI).A depressed lesion (0‐IIc) in the upper third of the stomach (13 mm in diameter) was observed. The final histopathological diagnosis was well‐differentiated adenocarcinoma, confined to the mucosa. The color difference was 5.7 in WLI, 19.1 in 3G‐NBI, and 2.4 in TXI

**FIGURE 6:**
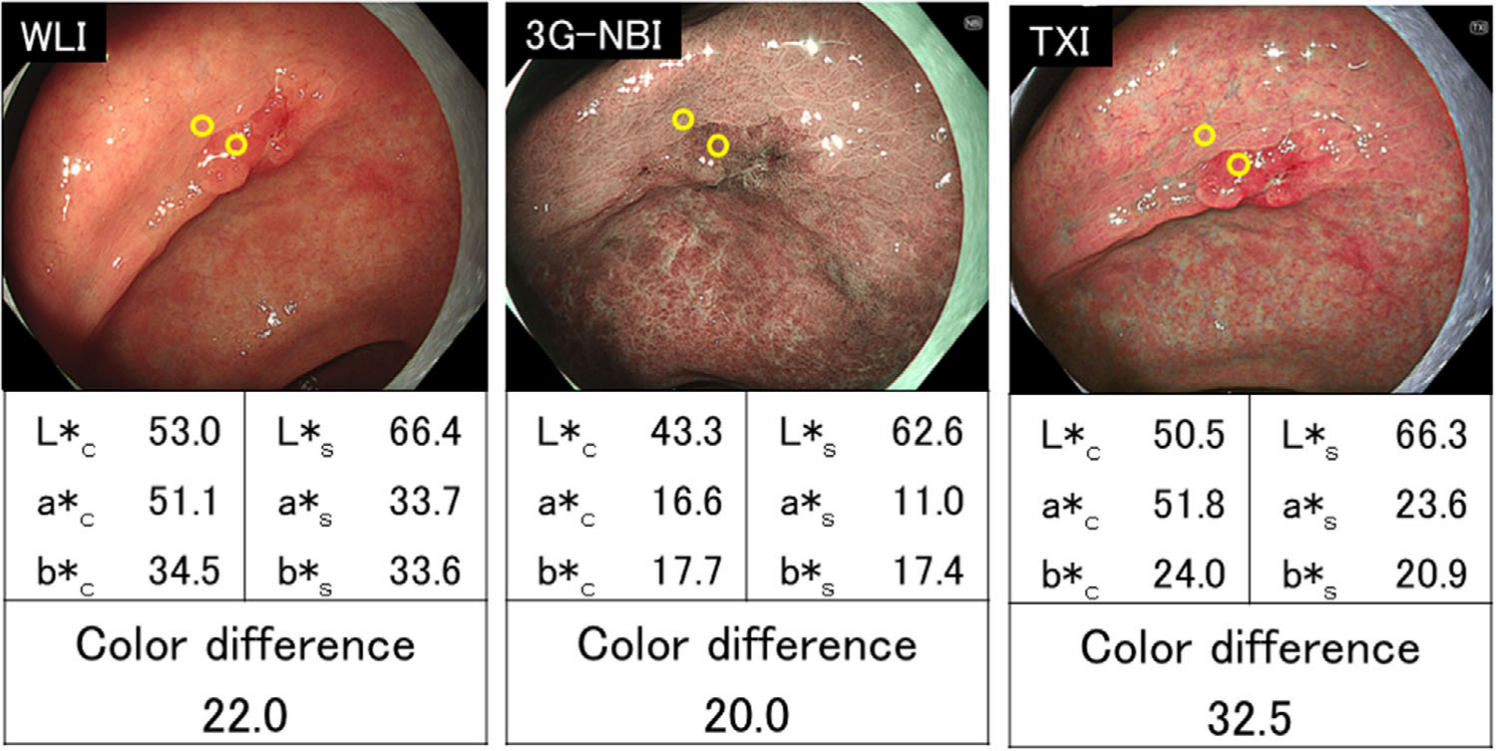
A representative case in which the color difference was larger in texture and color enhancement imaging (TXI) and smaller in third‐generation narrow band imaging (3G‐NBI) than in white light imaging (WLI). A depressed lesion (0–IIc) in the lower third of the stomach (25 mm in diameter) was observed. The final histopathological diagnosis was well‐differentiated adenocarcinoma, confined to the mucosa. The color difference was 22.0 in WLI, 20.0 in 3G‐NBI, and 32.5 in TXI

For each lesion, the color difference with respect to WLI is shown in Figure 4. The color difference in the two cases was smaller in 3G‐NBI and TXI than in WLI. The color difference in the six cases was larger in 3G‐NBI and smaller in TXI than in WLI (a representative case is shown in Figure 5). The color difference in the nine cases was larger in TXI and smaller in 3G‐NBI than in WLI (a representative case is shown in Figure 6).

The median color difference per background factor in each modality is presented in Table 2. The majority of background factors had large color differences for both TXI and 3G‐NBI compared with WLI. There were no background factors that showed significant differences in color differences between TXI and 3G‐NBI.

**TABLE 2:** Median color difference per background factors (*n* = 51)

	**Median color difference**			***p*‐Value**		
	WLI	3G‐NBI	TXI	WLI versus 3G‐NBI	WLI versus TXI	3G‐NBI versus TXI
Location						
upper–middle (*n* = 41)	9.9	13.6	16.1	<0.001	<0.001	0.280
lower (*n* = 10)	4.4	12.2	10.6	<0.001	<0.001	1.000
Macroscopic type						
0–IIa (*n* = 17)	8.9	11.9	15.3	<0.001	<0.001	0.404
0–IIb (*n* = 1)	3.6	7.5	3.0	1.000	1.000	1.000
0–IIc (*n* = 33)	9.8	15.1	15.6	<0.001	<0.001	0.560
Histological type						
Differentiated (*n* = 47)	9.4	13.6	15.3	<0.001	<0.001	0.626
Undifferentiated (*n* = 4)	8.5	12.7	16.5	0.250	0.125	0.250
Median tumor size						
<10 mm (*n* = 11)	7.7	13.4	13.2	<0.001	<0.001	0.638
≥10 mm (*n* = 40)	10.2	13.5	15.8	<0.001	<0.001	0.405
Invasion depth						
pT1a (*n* = 45)	8.3	13.4	15.1	<0.001	<0.001	0.388
pT1b (*n* = 6)	19.7	18.1	21.7	0.438	0.563	0.563
Endoscopic gastric atrophy						
Present (*n* = 49)	8.9	13.6	15.2	<0.001	<0.001	0.554
Absent (*n* = 2)	12.4	11.4	20.4	1.000	0.500	0.500
*H. pylori* infection status						
current/past (*n* = 49)	8.9	13.6	15.2	<0.001	<0.001	0.554
non‐infection (*n* = 2)	12.4	11.4	20.4	1.000	0.500	0.500
Gastrointestinal videoscope						
GIF‐EZ1500 (*n* = 28)	11.9	14.5	16.7	0.008	<0.001	0.186
GIF‐XZ1200 (*n* = 10)	7.2	9.4	9.0	0.010	0.020	0.432
GIF‐H290Z (*n* = 13)	8.9	13.6	17.0	<0.001	<0.001	0.685

Abbreviations: 3G‐NBI, third‐generation narrow band imaging; *H. pylori, Helicobacter pylori*; TXI, texture and color enhancement imaging; WLI, white light imaging.

## DISCUSSION

Our study showed that 3G‐NBI and TXI were more useful than WLI in improving the visibility of EGC in terms of color difference. However, there was no significant difference between 3G‐NBI and TXI. This is the first study to simultaneously investigate the usefulness of 3G‐NBI and TXI in improving the visibility of superficial EGC by evaluating the color difference.

To detect EGC in screening esophagogastroduodenoscopy using WLI, it is necessary to recognize color differences and morphological findings from the surrounding gastric mucosa. However, the endoscopic abnormalities observed in EGC are so slight that even expert endoscopists can miss EGC. IEE is expected to detect EGC more efficiently, and several studies have been conducted. Yoshida et al. reported that 2G‐NBI did not increase the EGC detection rate over WLI in an open‐label, randomized, controlled tandem trial (2G‐NBI: 2.3% vs WLI: 1.9%, *p* = 0.41).^9^ An endoscopic system using 5‐color LEDs was released worldwide in 2020, and we are now in the era of 3G‐NBI. However, no studies have examined the usefulness of 3G‐NBI for detecting EGC. TXI emphasizes three image components (texture, brightness, and color) in WLI, there were two previous studies that reported the visibility of EGC in TXI.^10^, ^11^ Ishikawa et al. investigated the color difference between non‐neoplastic and neoplastic areas of 12 gastric neoplasms in WLI and TXI.^10^ The color difference was significantly higher for TXI mode 1 than for WLI and TXI mode 2. Abe et al. also investigated the color difference between 20 non‐neoplastic and neoplastic areas of the stomach by comparing WLI, TXI mode 1, and TXI mode 2.^11^ Their study constructed still images of TXI mode 1 and TXI mode 2 from WLI images that were consistently in the distance, angle, and air insufflation, by using computer simulation. Their study demonstrated that TXI mode 1 could improve the visibility of EGC compared with WLI by more objective analysis than the study by Ishikawa et al. Both reports indicated that TXI may improve the visibility of EGC compared with WLI. However, none of the previous studies compared it with other IEEs.

We therefore simultaneously compared 3G‐NBI, TXI, and WLI, which are available for EGC screening. As a result, we were able to show the potential usefulness of 3G‐NBI and TXI for the detection of EGC. The majority of cases had larger color differences in both TXI and 3G‐NBI compared with WLI. Interestingly, however, only the color difference of either 3G‐NBI or TXI was larger than that of WLI in some cases. It is assumed that there are EGCs for which 3G‐NBI is more effective and EGCs for which TXI is more effective for detection. However, because of the small sample size, this issue could not be resolved in this study. Future large‐scale studies are expected.

The present study has several limitations. First, it was conducted at a single institution. Second, we examined visibility, not detectability. Since the results of this study do not indicate the usefulness of 3G‐NBI and TXI in actual clinical practice, the detection rate of EGCs will need to be studied in a multicenter, prospective, randomized, controlled trial. The third limitation is the identity of the image. For each lesion, we selected one non‐magnifying image for each method so that the location and size of the lesion in the three images were the same. Hence, the three images in each observation method are not exactly the same. Finally, since this study did not analyze the visibility of non‐cancerous lesions, false positives may increase in clinical practice. It is a matter to be considered in future clinical studies.

In conclusion, this investigation demonstrated that both 3G‐NBI and TXI were more useful than WLI in improving the visibility of EGC in terms of color difference.

## CONFLICT OF INTEREST

The authors declare that they have no conflict of interest.
